# Critical Evaluation of the Use of Cell Cultures for Inclusion in Clinical Trials of Patients Affected by Collagen VI Myopathies

**DOI:** 10.1002/jcp.23039

**Published:** 2011-09-27

**Authors:** P Sabatelli, E Palma, A Angelin, S Squarzoni, A Urciuolo, C Pellegrini, T Tiepolo, P Bonaldo, F Gualandi, L Merlini, P Bernardi, NM Maraldi

**Affiliations:** 1 CNR—National Research Council of Italy, Institute of Molecular Genetics c/o IORBologna, Italy; 2 Department of Biomedical Sciences, CNR Institute of Neuroscience, University of PadovaPadova, Italy; 3 Department of Histology, Microbiology and Medical Biotechnology, University of PadovaPadova, Italy; 4 Department of Experimental and Diagnostic Medicine, University of FerraraFerrara, Italy; 5 Laboratory of Musculoskeletal Cell Biology, Istituto Ortopedico RizzoliBologna, Italy; 6 Department of Anatomical Science, University of BolognaBologna, Italy

## Abstract

Collagen VI myopathies (Ullrich congenital muscular dystrophy (UCMD), Bethlem myopathy (BM), and myosclerosis myopathy) share a common pathogenesis, that is, mitochondrial dysfunction due to deregulation of the permeability transition pore (PTP). This effect was first identified in the *Col6a1*^*−/−*^ mouse model and then in muscle cell cultures from UCMD and BM patients; the normalizing effect of cyclosporin A (CsA) confirmed the pathogenic role of PTP opening. In order to determine whether mitochondrial performance can be used as a criterion for inclusion in clinical trials and as an outcome measure of the patient response to therapy, it is mandatory to establish whether mitochondrial dysfunction is conserved in primary cell cultures from UCMD and BM patients. In this study we report evidence that mitochondrial dysfunction and the consequent increase of apoptotic rate can be detected not only, as previously reported, in muscle, but also in fibroblast cell cultures established from muscle biopsies of collagen VI-related myopathic patients. However, the mitochondrial phenotype is no longer maintained after nine passages in culture. These data demonstrate that the dire consequences of mitochondrial dysfunction are not limited to myogenic cells, and that this parameter can be used as a suitable diagnostic criterion, provided that the cell culture conditions are carefully established.

J. Cell. Physiol. 227: 2927–2935, 2012. © 2011 Wiley Periodicals, Inc.

The extracellular matrix (ECM) plays a critical role in maintaining muscle function and integrity, and defects of its constituent proteins such as laminin-2 and collagen (Col) VI are involved in the molecular pathogenesis of various forms of muscular dystrophy (Schessl et al., 2006). ColVI is a major ECM protein forming a distinct microfilamentous network in several organs including skeletal muscle (Keene et al., 1988). In this tissue ColVI is a major component of the endomysium, where it is localized just outside the basement membrane (Kuo et al., 1997). ColVI controls survival and proliferation of normal and transformed cells, and increased levels of ColVI are associated with tumorigenesis and resistance to chemotherapeutic drugs (Iyengar et al., 2003; Sherman-Baust et al., 2003).

Deficiency of ColVI in humans due to mutations of *COL6* genes gives rise to three main muscle disorders, Bethlem myopathy (BM, MIM #158810), Ullrich congenital muscular dystrophy (UCMD, MIM #254090) (Lampe and Bushby, 2005), and myosclerosis myopathy (MIM #255600) (Merlini et al., 2008b).

BM is characterized by axial and proximal muscle weakness (Bethlem and Wijngaarden, 1976), and the hallmark of the disease is the presence of contractures of the interphalangeal joints of the last four fingers (Merlini et al., 1994). BM is a very heterogeneous disorder and patients show a wide range of clinical features, from mild myopathy to more severe cases with early onset and features of progressive muscular dystrophy (Jöbsis et al., 1999). Immunohistochemistry shows apparently normal or mildly reduced levels of ColVI in the endomysium of most BM patients.

UCMD is a severe congenital muscular dystrophy characterized by early onset, generalized and rapidly progressive muscle wasting and weakness, proximal joint contractures, and distal joint hyperflexibility. Walking ability is rarely achieved or preserved in UCMD patients, and the rapid progression of the clinical symptoms usually leads to early death, due to respiratory failure (Camacho Vanegas et al., 2001; Demir et al., 2002). ColVI is usually strongly reduced or absent in the muscle endomysium of UCMD patients. Cultured skin fibroblasts of UCMD patients show either a markedly decreased secretion of ColVI or lack of the characteristic filamentous network in the ECM, suggesting that UCMD mutations severely affect the synthesis and secretion of ColVI (Camacho Vanegas et al., 2001; Zhang et al., 2002; Squarzoni et al., 2006).

Myosclerosis Myopathy is characterized by progressive contractures of all joints, including jaws, spine, shoulders, elbows, wrists, fingers, hips, and knees. Patients are affected by scoliosis, mild girdle and proximal limb weakness, and moderate distal weakness. Muscles become thin and sclerotic and reach a woody consistency, causing diffuse restriction of movement of all joints, with marked difficulty in running and climbing stairs (Merlini et al., 2008b).

About 70 different mutations of the *COL6* genes have so far been associated either with UCMD or with BM (Lampe and Bushby, 2005); and although the reason why certain *COL6* mutations cause BM and others cause UCMD remains obscure, it appears that these disorders represent a clinical continuum rather than strictly separate entities (Pepe et al., 2002; Lampe and Bushby, 2005).

On the other hand, ColVI myopathies appear to share a common pathogenesis, that is, mitochondrial dysfunction due to deregulation of the permeability transition pore (PTP), an inner membrane high conductance channel (Bernardi et al., 2006). First identified in the *Col6a1*^*−/−*^ mouse model (Irwin et al., 2003), the mitochondrial defect has been detected in cultures from UCDM and BM patients (Angelin et al., 2007; Angelin et al., 2008), findings that led to a promising pilot trial with the PTP inhibitor cyclosporin A (CsA) (Merlini et al., 2008a). The normalizing effect of CsA (Irwin et al., 2003), of its non-immunosuppressive derivative Debio 025 (Tiepolo et al., 2009), as well as of genetic inactivation of the *Ppif* gene encoding cyclophilin (CyP) D (the mitochondrial receptor for CsA) in the *Col6a1*^*−/−*^ mouse (Palma et al., 2009) confirmed the pathogenic role of PTP opening, whose consequences are amplified by defective autophagy of the abnormal mitochondria (Grumati et al., 2010).

A typical feature of primary muscle-derived cultures from *Col6a1*^*−/−*^ mice, UCMD, and BM patients is abnormal depolarization induced by the F1FO ATP synthase inhibitor oligomycin (Klöhn et al., 2003; Angelin et al., 2007; Angelin et al., 2008; Merlini et al., 2008a), a phenotype that is normalized by treatment with CyP inhibitors, plating on ColVI, CyPD ablation, or stimulation of autophagy (Irwin et al., 2003; Angelin et al., 2007; Merlini et al., 2008a; Tiepolo et al., 2009; Palma et al., 2009; Grumati et al., 2010).

In this study we report evidence that mitochondrial dysfunction and the consequent increase of apoptotic rate occurs in cell cultures derived from muscle biopsies of UCMD and BM patients, many of which were not included in our previous studies. We provide evidence that the mitocondrial dysfunction occurs not only, as previously reported, in muscle, but also in fibroblast cell cultures established from muscle biopsies of these patients. Furthermore, the reported data indicate that the mitochondrial phenotype is no longer maintained after nine passages in culture. These data demonstrate that the dire consequences of mitochondrial dysfunction are not limited to myogenic cells, and that this parameter can be used as a suitable diagnostic criterion for inclusion in clinical trials and for evaluating the response to therapy, provided that the cell culture conditions are carefully established.

## Materials and Methods

### Patients

BM and UCMD were diagnosed according to the criteria of the European Neuromuscular Center (Pepe et al., 2002). All patients were examined and all underwent a muscle biopsy. The basic features of the patients analyzed in this study are summarized in Table 1. All participants provided written informed consent, and approval was obtained from the Ethics Committee of the Rizzoli Orthopedic Institute (Bologna, Italy).

**TABLE 1:** Features of patients involved in the study

Patient no.	Phenotype	Age (year)	Collagen VI	Mutation
1	BM, walker	31	Normal in skin, cultured fibroblasts, and skeletal muscle	COL6A3: heterozygous Gly2077Asp
2	BM, walker	57	Normal in muscle fibers and decreased in fibroblasts	COL6A1: heterozygous Tyr122–Gly143del
3	BM, walker	7	Normal in skin, cultured fibroblasts, and mildly reduced in skeletal muscle	No mutations in COL6A1, COL6A2, COL6A3
4	BM, walker	17	Normal in skin, cultured fibroblasts, and skeletal muscle	COL6A2: heterozygous Gly700Ser
5	UCMD, non-walker	6	Marked reduction in cultured skin fibroblasts and in muscle fibers	COL6A2: compound heterozygous Gly487Ala495delAspfsX48 and Glu591Cys605delThrfsX148

### Primary muscle-derived cell cultures

We obtained muscle biopsies from healthy donors, BM, and UCMD patients. Cell cultures were established using enzymatic and mechanical treatment and plating in Dulbecco's modified Eagle's medium (DMEM) plus 20% fetal calf serum (FCS) and antibiotics (penicillin, streptomycin, and amphotericin B; Sigma, St. Louis, MO) and then stored in liquid nitrogen.

### Immunofluorescence on cell cultures

Muscle-derived cell cultures were fixed with cold methanol, washed with PBS, and incubated with desmin antibody (Abcam, Cambridge, UK). The immunoreaction was revealed with TRITC-conjugated antibody (DAKO, Atlanta, GA) and the samples were mounted with Pro-long anti-fade reagent (Molecular Probes, Eugene, OR) and examined with Nikon epifluorescence microscope at ×100 magnification.

### Measurements of mitochondrial membrane potential

Mitochondrial membrane potential was measured by epifluorescence microscopy based on the accumulation of tetramethylrhodamine methyl ester (TMRM) as described previously (Angelin et al., 2007). Muscle-derived cell cultures (30,000–35,000 cells) were seeded onto 24 mm-diameter round glass coverslips, grown for 2 days in DMEM supplemented with 20% FCS and studied as described after loading with 10 nM TMRM in 1 ml of serum-free DMEM.

### Transmission electron microscopy

Muscle-derived cells were grown in DMEM supplemented with 20% FCS until confluence, fixed with 2.5% glutaraldehyde in cacodylate buffer 0.1 M, pH 7.2, and post-fixed with 1% osmium tetroxide in cacodylate buffer 0.1 M, pH 7.3. After detaching with propylene oxide, the cells were centrifuged and embedded in Epon 812. Utrathin sections were stained with uranyl acetate and lead citrate and observed with a Philips EM400 operating at 100 kV.

### Scanning electron microscopy back-scattered imaging

Cu–Pb staining solution was prepared by mixing 20 ml of 4.6% trisodium citrate, 2 ml of 33% lead nitrate, and 0.5 ml of 10% copper sulfate (pH 9), as previously described (LeFurgey et al., 1983), and used undiluted. Cells were fixed with 2.5% glutaraldehyde–0.1 M cacodylate buffer pH 7.3 for 1 h at 4°C. After washing with 0.1 M cacodylate buffer pH 7.3, the coverslips were brought to room temperature, washed three times with distilled water and incubated with Cu–Pb solution at 4°C overnight. The samples were then brought to room temperature, washed three times with distilled water and post-fixed with 1% osmium tetroxide in 0.1 M cacodylate buffer for 1 h at room temperature. After rinsing with 0.1 M cacodylate buffer and then with distilled water, the samples were dehydrated in an ethanol series and critical point dried. The coverslips carrying cells were then mounted on aluminum stubs with silver adhesive paint, coated with spectrographic carbon by vacuum evaporation in an Ewards E 306 apparatus, and observed at 0° tilt angle with a Cambridge Stereoscan 200 scanning electron microscope equipped with an Oxford Tetra solid state BSE detector. Acceleration voltage was set at 30 kV and WD at 15 mm.

### TUNEL and immunofluorescence analysis

TUNEL assays were performed with the Dead End Fluorometric in situ apoptosis detection system (Promega Italia, Milan, Italy). Muscles cultures were permeabilized in methanol–acetone 50:50 at −20°C for 10 min. After drying, slides were washed in PBS and processed for immunofluorescence with an antibody against desmin (D1033, Sigma). Samples were washed and then incubated with equilibration buffer for 10 min and treated with buffer containing fluorescent nucleotides, rTdT enzyme, and Hoechst 33258 (Sigma) for 1 h at 37°C. SSC solution was used to block the activity of rTdT enzyme, before washing and preparing slides for microscopy analysis.

## Results

We tested the effects of oligomycin on the accumulation of TMRM by mitochondria in cell cultures established from muscle biopsies of one UCMD patient and of four patients affected by BM (Table 1). Among these last cases, while one of them has been included in a study previously reported (Angelin et al., 2008), three are new cases, two of which having mutations in one of the COL6 gene, and one with no mutations in none of the COL6 genes. In BM patients 1, 2, and 4, ColVI expression was normal in both muscle fibers and in cultured skin fibroblasts, while in BM patient 3 a mild reduction was detected only in muscle biopsy. In UCMD patient, a marked ColVI reduction was detected in both muscle biopsy and fibroblast culture.

Cell cultures derived from muscle biopsies are heterogeneous, and may contain variable proportions of myoblasts and interstitial fibroblasts. Therefore, we analyzed the relative percentage of desmin-positive cells at different passages of primary cell cultures derived from muscle biopsies of a normal donor, a UCMD patient, and two BM patients (Table 2). A significant, progressive decrease of desmin-positive cells occurs in control as well as in the patient samples, although the initial percentage of myoblasts is highly variable in the patient samples with respect to control. In fact, at passage five, the percentage of myoblasts is highly reduced in all cases. A further significant reduction is detected at passage 12 except for the UCMD patient in which the value was not significantly further decreased.

**TABLE 2:** Percentage ± SD of desmin-positive cells in cell cultures from muscle biopsies of patients and control subject

Cell culture	Passage 0	Passage 5	Passage 12
Control	27.0 ± 3.6	7.0 ± 2.6^a^	1.2 ± 0.7^a^
Patient 1	25.0 ± 2.6	0.7 ± 0.5^a^	0.2 ± 0.1^a^
Patient 2	12.0 ± 3.0	3.0 ± 0.9^a^	1.9 ± 0.6^a^
Patient 5	29.6 ± 2.2	5.8 ± 1.6^a^	5.5 ± 1.3

a Significance calculated by Mann–Whitney test *P* < 0.01.

These data indicate that the cells populations present in a muscle biopsy are differently represented, depending on the disease progression. Furthermore, the percentage of myoblasts is progressively reduced by maintaining cells in culture conditions and by increasing the number of passages. In any case, the main population is constituted by stromal fibroblasts, which substantially represent the bulk of cells at high passages cultures.

**Fig. 1:**
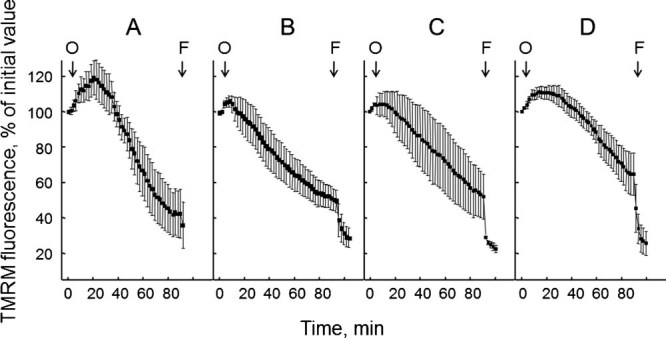
Mitochondrial response to oligomycin in muscle-derived cultures from BM patients. Cells from the four patients affected by BM described in Table 1 and passaged less than 7 times in culture were loaded with TMRM as described in the Materials Methods Section. When indicated by arrows, 6 µM oligomycin (O) and 4 µM FCCP (F) were added. Each part refers to one individual patient, and reported values are the mean ± SEM of at least four experiments.

In keeping with our previous results on one such patient (Angelin et al., 2007), in all cases the probe was released from mitochondria (Fig. 1), consistent with the existence of a latent mitochondrial dysfunction that can be precipitated by inhibition of the F_1_F_O_ ATP synthase, possibly a result of Ca^2+^ deregulation (Angelin et al., 2008). Since skin fibroblasts from ColVI myopathic patients were reported not to respond with depolarization to oligomycin (Hicks et al., 2009), we investigated whether mitochondrial dysfunction is restricted to myogenic cells.

**Fig. 2:**
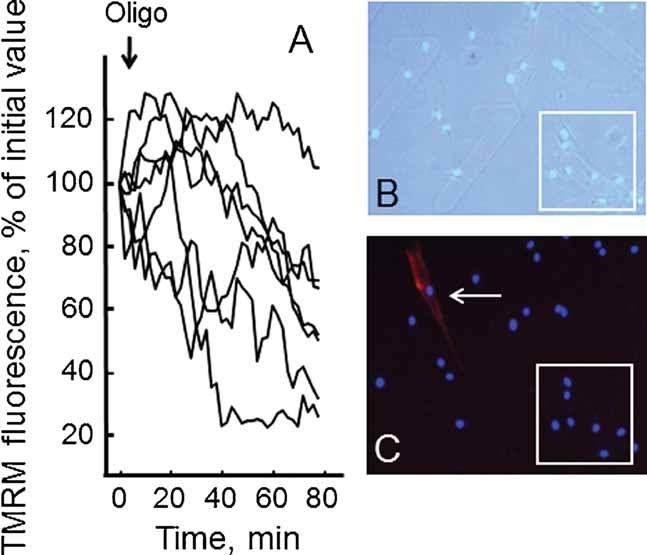
Analysis of the mitochondrial response to oligomycin in desmin-negative cells from one BM patient. Cells from one BM patient passaged less than seven times in culture were loaded with TMRM as described in the Materials Methods Section. Part A: Response of the mitochondrial membrane potential as measured from TMRM fluorescence after the addition of 6 µM oligomycin (Oligo), where the traces report recordings from the region boxed in Parts (B) and (C). Part B: Phase contrast image of cells after fixation and staining for desmin and DAPI, which was used to identify the cells previously analyzed for TMRM fluorescence. Part C: Fluorescence image of the same cells of part (B), which allows identification of desmin-positive (red) cells, one of which is visible in this field (arrow).

**Fig. 3:**
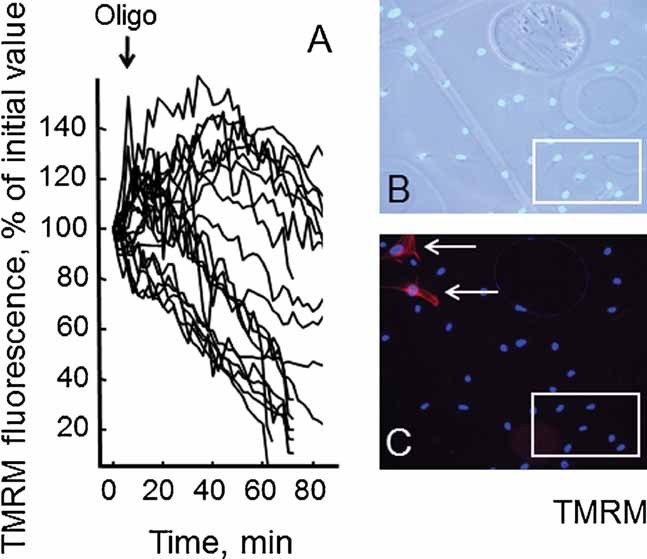
Analysis of mitochondrial response to oligomycin in desmin-negative cells from the UCMD patient. Cells from the UCMD patient described in Table 1 passaged less than seven times in culture were loaded with TMRM as described in the Materials Methods Section. Conditions were otherwise identical to those described in Fig. 2.

Primary muscle cultures from a muscle biopsy of a BM patient were grown on gridded coverslips, so that the position of individual cells could be tracked, and subjected to treatment with oligomycin, which caused the expected depolarization in a randomly selected group of cells (Fig. 2A). Cells were then fixed, treated with DAPI, and the cells previously monitored for TMRM fluorescence identified (boxed area in Fig. 2B,C). Immunostaining for desmin revealed that only rare cells were positive for this myogenic marker (one in the area shown in Fig. 2C, arrow). On the other hand, all oligomycin-responsive cells were desmin-negative, a result that was fully confirmed in a similar experiment carried out in cultures from the UCMD patient (Fig. 3).

**Fig. 4:**
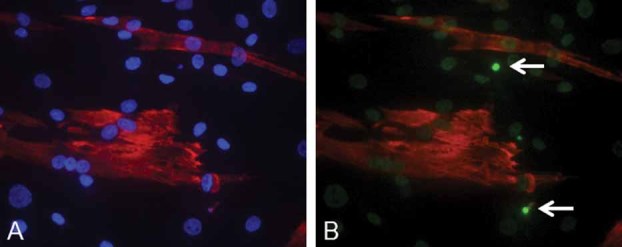
Occurrence of apoptosis in cells from the muscle biopsy of the UCMD patient. Representative image of the immunohistochemical analysis performed on early-passage cell cultures (less than seven passages) established from muscle biopsies of the UCMD patient treated with an antibody against desmin (red) and counterstained with Hoechst (part A) and subjected to the TUNEL reaction (part B, two positive nuclei marked by arrows).

These results indicate that muscle fibroblasts from BM and UCMD patients have a latent mitochondrial dysfunction that can be triggered by oligomycin (Merlini et al., 2008a). Importantly, desmin-negative cells also displayed apoptotic nuclei as detected by the TUNEL reaction (Fig. 4), indicating that the dire consequences of mitochondrial dysfunction are not limited to myogenic cells.

**Fig. 5:**
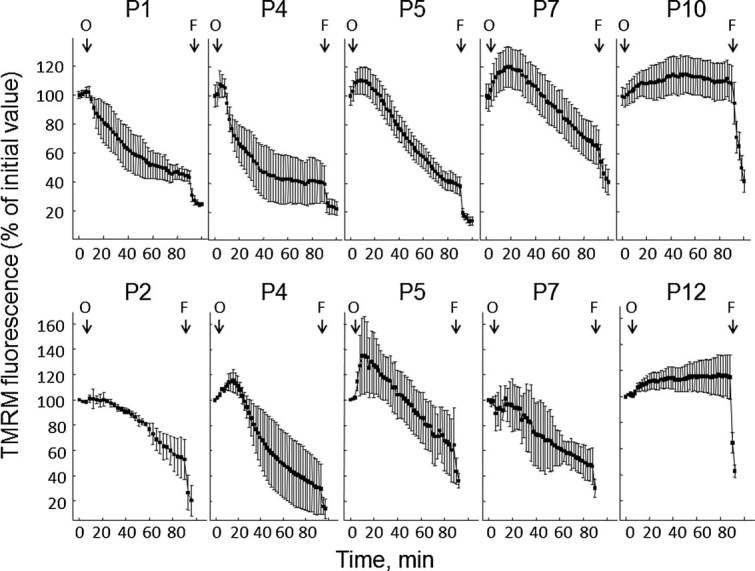
Analysis of the mitochondrial response to oligomycin in cells derived from one BM and one UCMD patient after different passages in culture. Cultures established from muscle biopsies of one patient with BM and one patient with UCMD were tested for the mitochondrial response to oligomycin after the indicated number of culture. Where indicated 6 µM oligomycin (O) and 4 µM FCCP (F) were added. Each trace represents the mean ± SEM of at least three experiments.

Mitochondrial dysfunction, often caused by opening of the PTP, is causally involved in cell death (Bernardi et al., 2006). We reasoned that in vitro passage of cultures might be selecting cells that are more resistant to death. We therefore tested cultures from one BM and one UCMD patient for their mitochondrial response to oligomycin as a function of the number of passages. For both cell cultures the expected depolarizing response to oligomycin was consistently observed up to the 7th passage, while it disappeared at higher passage numbers, a result that we have confirmed with all the cell cultures of the present study although the full time-course was performed only for the two cases shown in Figure 5.

**Fig. 6:**
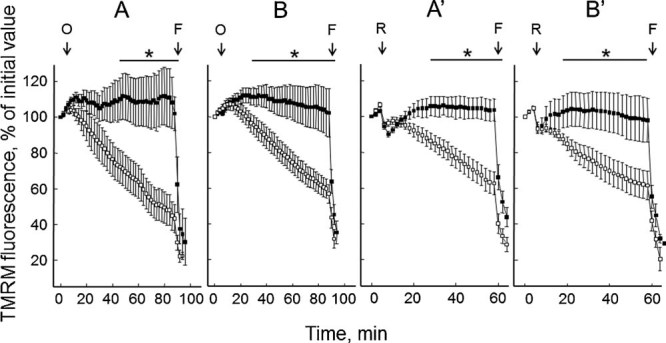
Analysis of the effect of oligomycin and rotenone on mitochondrial membrane potential in cells from one BM (parts B and B') and the UCMD (parts A and A') patient below 8 and above 9 passages. Parts A, A': BM patient. Parts B, B': UCMD patient. Open symbols, cells passaged up to seven times; closed symbols, cells passaged more than nine times. Where indicated 6 µM oligomycin (O) or 2 µM rotenone (R) and 4 µM FCCP (F) were added. For each patient, the graph reports the mean of cells tested passages at least ± SEM. The bar marked by an asterisk denotes a *P* < 0.05 for the two sets passages (P) in at four different of data points.

**Fig. 7:**
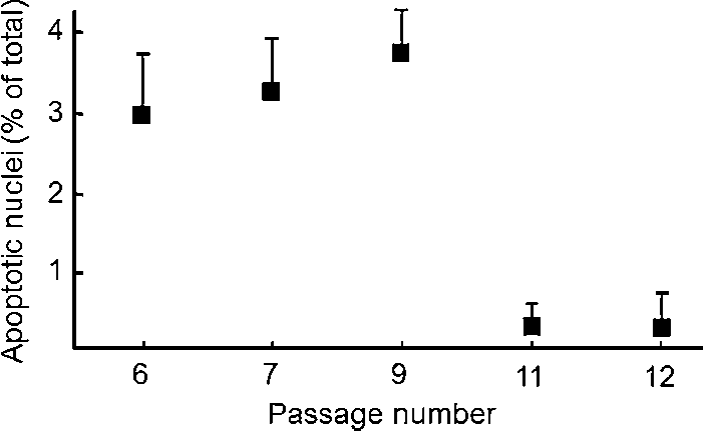
Incidence of apoptosis in muscle-derived cell culture from the UCMD patient as a function of the number of passages in culture. The percentage of apoptotic nuclei was determined by the TUNEL assay as described in the Materials Methods Section. Data are the mean of at least four experiments ± SEM and represent the level of apoptosis in cultures at the indicated number of cell passages.

A statistical analysis was performed by pooling the results obtained from measurements performed on cells passages up to seven times, or more than nine. The difference was striking and highly significant (Fig. 6A,B). We tested if a similar resistance could be observed in response to rotenone, which also causes depolarization of mitochondria in cells from UCMD patients presumably because of the altered voltage threshold of the PTP (Angelin et al., 2008). Also in the case of treatment with rotenone depolarization was observed with early- but not late-passage cultures from both the BM (Fig. 6A') and the UCMD patient (Fig. 6B'). The striking selection of depolarization-resistant cells was matched by an abrupt decrease of the incidence of apoptotic nuclei after 11 passages (Fig. 7), a result that is reminiscent of that obtained by plating cells from ColVI myopathy patients on ColVI, or by treating them with CsA (Angelin et al., 2007).

**Fig. 8:**
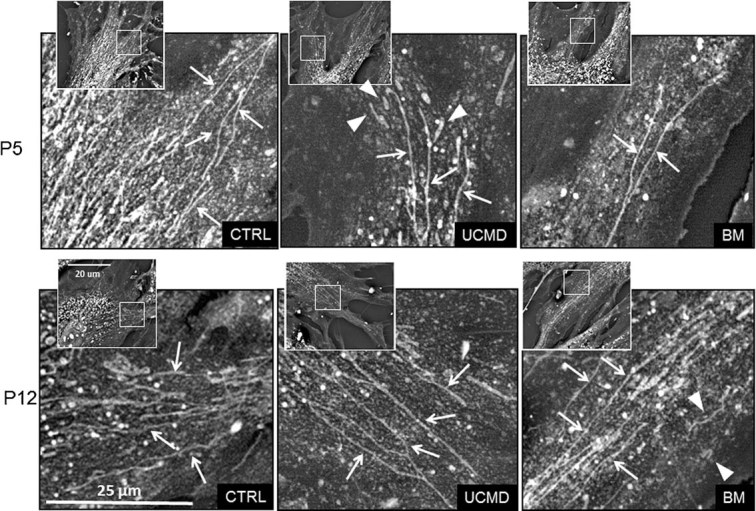
Back-scattered imaging of muscle-derived cell cultures from BM and UCMD patients at different culture passages. Arrows point at long and branched mitochondria; and arrowheads at fragmented/swollen mitochondria. In samples at P5, UCMD, and BM cells display a less developed mitochondrial network compared to samples at P12 and to control cells at P5. In samples at P12, UCMD, and BM cells show a more developed mitochondrial network similar to healthy control. Fragmented and swollen mitochondria appear more frequently in patients' cells. Insets: Low magnification view of the same cells; squared areas indicate the magnified areas. P, culture passage; UCMD, Ullrich congenital muscular dystrophy; BM, Bethlem myopathy.

**Fig. 9:**
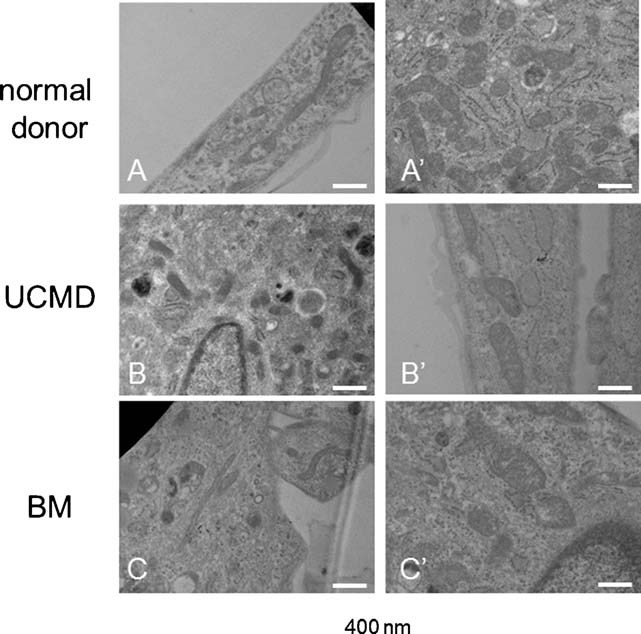
Transmission electron microscopy analysis of Epon-embedded cell cultures from BM and UCMD patients at different culture passage. At low passage number (A–C), altered mitochondria characterized by reduced short axis are visible in both UCMD and BM patient cultures (arrows). At high passages, the mitochondria of both patients show a morphology very similar to that of normal control. Scale bar, 400 nm.

We finally investigated whether the resistant cells also had a lower incidence of mitochondrial ultrastructural abnormalities, which are characteristic of the mouse *Col6a1*^*−/−*^ model (Irwin et al., 2003) and can also be observed in cultures established from muscle biopsies of UCMD and BM patients (Angelin et al., 2007). Back scattered imaging of cultured cells allowed to clearly monitor both organization and extension of the mitochondrial reticulum by exploiting the specific staining of mitochondrial membranes with copper-lead salts solutions (Thiéry, 1973). At low passage number, both UCMD (patient 5) and BM (patient 2) cells showed reduced mitochondrial branching, and some fragmented and swollen mitochondria were detected. At high passage numbers, the mitochondrial reticulum appeared more developed and similar to controls as long, thin and branched mitochondria were detected (Fig. 8). Mitochondrial abnormalities were also detected in thin sections of UCMD and BM patient cells when examined at low passage number. The most relevant alterations consisted in an increased mitochondrial area/perimeter ratio (72% in UCMD and 41% in BM muscle cell cultures, higher than those of an unaffected donor with *P* < 0.05) and reduced short axis (between 90 and 120 nm in BM and UCMD patients, and >200 nm in unaffected donor). Rare swollen mitochondria as well as megaconial aspects were also detected in cultures of both patients. Remarkably, the area/perimeter ratio and short axis values at passage 12 were instead similar to those of unaffected donor at the same passage number (Fig. 9).

## Discussion

Interstitial fibroblasts from muscle biopsies could represent a resource for diagnostic tests in UCMD and BM patients, because these cells have been reported to be the main source of ColVI in skeletal muscle (Zou et al., 2008). It is therefore important to assess whether the mitochondrial membrane potential and its response to oligomycin and rotenone can be used as a suitable index of mitochondrial latent dysfunction in cell cultures obtained from patient muscular biopsies.

Given the heterogeneity of cell cultures derived from muscle biopsies, the principal aim of this study was to determine to which extent, they can be utilized to determine mitochondrial dysfunction in ColVI-related myopathies.

In previous studies, a latent mitochondrial dysfunction that can be unmasked by the addition of oligomycin has been measured based on the response of mitochondrial TMRM in the *Col6*^*−/−*^ mouse model (Irwin et al., 2003). The mitochondrial defect could also be observed in isolated myofibers freshly prepared from muscle biopsies of *Col6*^*−/−*^ mice and in muscle cultures of UCMD patients; the mitochondrial defect and increased rate of apoptosis could be rescued by plating cells on purified ColVI or by treatment with CsA (Irwin et al., 2003; Angelin et al., 2007). Similar results have been obtained in a clinical study on five patients, in which cells derived from a muscular biopsy before and after 20 day treatment with CsA have been studied (Merlini et al., 2008a). In these conditions, the primary cell cultures contained 25–35% desmin-positive cells, while the majority of the other desmin-negative, vimentin-positive cells, conceivably represent interstitial muscle fibroblasts (Bernardi et al., 2009). It is well known that maintenance in culture of muscle-derived cells progressively causes a reduction of myoblasts, so that after 5–7 passages, the percentage of desmin-positive cells is highly reduced, possibly due to the higher proliferation rate of fibroblasts. On the other hand, fibroblasts in muscle are a key mechanistic component in ColVI-related myopathies, because interstitial fibroblasts play a role in prepatterning muscle development (Kardon et al., 2003) and are involved in the synthesis and arrangement of the ECM and of ColVI in muscle (Engvall et al., 1986; Zou et al., 2008). Interestingly, the retention of ColVI in interstitial fibroblasts but not in myocytes in biopsies of patients with known ColVI-retaining mutations, point to fibroblasts as the cells to be analyzed in the culture systems (Zou et al., 2008).

Mutant ColVI is retained in dermal fibroblasts from a UCMD patient (Pan et al., 2003) and in dermal fibroblasts in culture obtained from BM and UCMD patients (Zou et al., 2008). Therefore, it has been inferred that skin biopsy-derived fibroblasts should be utilized for diagnostic use in ColVI-related myopathies (Hicks et al., 2008).

In a recent study of mitochondrial dysfunction and its rescue, PTP dysregulation has been analyzed by utilizing skin fibroblasts from biopsies of a group of patients and muscle-derived cell cultures obtained from a Tissue Culture Collection (Hicks et al., 2008). A result of this study indicated that mitochondrial depolarization inducible by oligomycin is not common in skin biopsy-derived fibroblasts cells of BM and UCMD patients, being present only in a case showing a complete absence of intracellular and extracellular ColVI (Hicks et al., 2008). This finding suggests that skin fibroblasts may not display mitochondrial dysfunction, which is instead present in their muscle-derived, desmin-negative muscle fibroblasts, suggesting that great care should be exerted in utilizing these samples to monitor mitochondrial dysfunction. A further result of the same investigation indicated that mitochondrial depolarization was present in UCMD muscle-derived cell cultures but not in fibroblast cultures from the same patient (Hicks et al., 2008). The interpretation of this result deserves a critical analysis, because whether or not a mitochondrial phenotype can be detected in cells from a patient biopsy is an important issue for inclusion in clinical trials. In this study we have addressed this problem experimentally.

Our results show that latent mitochondrial dysfunction is present both in muscle cells and in muscle fibroblasts derived from muscle cultures of UCMD and BM patients. These results confirm that mitochondrial dysfunction is a key feature of ColVI-related diseases. Moreover, the persistence of a mitochondrial dysfunction in cell cultures derived from myopathic patients may have a key diagnostic significance.

The mitochondrial dysfunction has been documented in three BM patients, not included in our previous studies; interestingly, two of these patients present mutations in one of the ColVI chains, while one patient does not present ColVI-related mutations. However, in this patients, whose genetic profile is under investigation, ColVI expression appears to be reduced, possibly owing to a secondary effect of a not yet identified genetic defect.

This suggests that the pathogenic mechanism leading to mitochondrial dysfunction could be effective also in other myopathies; this possibility has been recently confirmed in a study where oligomycin-induced mitochondrial depolarization has been observed also in cells derived from a patient affected by limb-girdle muscular dystrophy 2B (Hicks et al., 2009).

Our current findings confirm the importance of mitochondria in the pathogenesis of ColVI muscular dystrophies and their role as potential targets for therapy (Angelin et al., 2007). Additional evidence for the mitochondrial pathogenesis of ColVI muscular dystrophies came from recent work in zebrafish, an organism where the PTP has regulatory features indistinguishable from those of mammals (Azzolin et al., 2010). Injection of morpholino to exon 13 of *col6a1* caused an in-frame deletion in the C-terminal part of the ColVI triple helical domain, a typical dominant BM mutation (Lucioli et al., 2005; Lampe et al., 2008) and resulted in a mild myopathy with late-onset motor deficits and obvious histopathologic abnormalities (Telfer et al., 2010); while injection of morpholino to exon 9 of *col6a1* caused an in-frame deletion in the N-terminal part of the triple helical domain, a dominant mutation in UCMD (Pepe et al., 2006; Lampe et al., 2008) and resulted in the predicted severe myopathy with early-onset motor deficits and severe ultrastructural changes (Telfer et al., 2010). In both cases mitochondrial abnormalities and increased apoptosis were observed, and treatment with CsA improved these pathological signs together with the motor deficit (Telfer et al., 2010).

Here we reported that the mitochondrial dysfunction detectable in cultured cells from ColVI-related myopathies is conserved in cultures at low passage number. On the other hand, in cell cultures at high passage number mitochondrial function appeared to be normalized, as assessed on the lack of depolarization by both oligomycin and rotenone and on recovery of a normal morphology by both transmission and scanning electron microscopy with back-scattered imaging. Remarkably, apoptotic levels also dropped dramatically, supporting a cause–effect relationship between mitochondrial function and cell survival.

It is well known that cell cultures obtained from muscle biopsies are heterogeneous, including myoblasts, muscular fibroblasts, myofibroblasts, and rare mesenchimal stem cells; all these cells may undergo selection/modification processes during passage in culture.

Since the relative amount of the two cell populations changes along the observed period of culture, the selection of a cell population seems to occur. The percentage of desmin-positive cells, in fact, decreases relative to that of desmin-negative cells in late passages, probably because muscular fibroblasts proliferate at a higher rate compared to myoblasts.

It is well known that cells from ColVI deficient mice and UCMD/BM patients present a “latent” mitochondrial dysfunction, which can be unmasked by oligomycin. It is thus quite possible that, in cell culture condition, cells having only a reduced percentage of damaged mitochondria may survive, so that, after a given number of passages, the triggering effect of oligomycin on latent mitochondrial depolarization is lost.

In conclusion, our data indicate that the mitochondrial response to oligomycin and rotenone can be used as a criterion for inclusion in clinical trials and as an outcome measure of the patient response to therapy (Merlini and Bernardi, 2008; Merlini et al., 2008a). However, the observed decrease of response to these drugs suggests that cell cultures obtained from muscle biopsies can be utilized at low-passages only, because the permanence in cell culture appears to select resistant cell populations through mechanisms that require further investigation.

## Abbreviations:

BM: Bethlem myopathy
Col: collagen
CyP: cyclophilin
CsA: cyclosporin A
ECM: extracellular matrix
PTP: permeability transition pore
TMRM: tetramethylrhodamine methyl ester
UCMD: Ullrich congenital muscular dystrophy
